# Syntheses and structures of two benzoyl amides: 2-chloro-4-eth­oxy-3,5-dimeth­oxy-*N*-(3-oxo­cyclo­hex-1-en-1-yl)benzamide and 2-chloro-*N*-(5,5-dimethyl-3-oxo­cyclo­hex-1-en-1-yl)-4-eth­oxy-3,5-di­meth­oxy­benzamide

**DOI:** 10.1107/S2056989021001778

**Published:** 2021-02-26

**Authors:** Alan J. Anderson, Ray J. Butcher, Edward Ollie

**Affiliations:** a Department of Natural Sciences, Bowie State University, 14000 Jericho Park Road, Bowie, MD 20715-9465, USA; b Department of Chemistry, Howard University, 525 College Street NW, Washington DC 20059, USA

**Keywords:** crystal structure, enamino­nes, bioactivity

## Abstract

The first title benzoyl amide, C_17_H_20_ClNO_5_ (**3a**), crystallizes in the monoclinic space group *P*2_1_/*c* with *Z* = 4 and the second, C_19_H_24_ClNO_5_ (**3b**), also crystallizes in *P*2_1_/*c* with *Z* = 8 (*Z*′ = 2), thus there are two independent mol­ecules in the asymmetric unit. In **3a**, the phenyl ring makes a dihedral angle of 50.8 (3)° with the amide moiety with the C=O group on the same side of the mol­ecule as the C—Cl group. One meth­oxy group is almost in the plane of the benzene ring, while the eth­oxy and other meth­oxy substituent are arranged on opposite sides of the ring with the eth­oxy group occupying the same side of the ring as the C=O group in the amide moiety. For one of the two mol­ecules in **3b**, both the amide and 5,5-dimethyl-3-oxo­cyclo­hex-1-en-1-yl moieties are disordered over two sets of sites with occupancies of 0.551 (2)/0.449 (2) with the major difference between the two conformers being due to the conformation adopted by the cyclo­hex-2-en-1-one ring. The three mol­ecules in **3b** (*i.e.*, the undisordered mol­ecule and the two disorder components) differ in the arrangement of the subsituents on the phenyl ring and the conformation adopted by their 5,5-dimethyl-3-oxo­cyclo­hex-1-en-1-yl moieties. In the crystal of **3a**, N—H⋯O hydrogen bonds link the mol­ecules into a zigzag chain propagating in the [001] direction. For **3b** a combination of C—H⋯O and N—H⋯O inter­molecular inter­actions link the mol­ecules into a zigzag ribbon propagating in the [001] direction.

## Abstract 2

The crystal structures of two benzoyl amides: 2-chloro-4-eth­oxy-3,5-dimeth­oxy-*N*-(3-oxo­cyclo­hex-1-en-1-yl)benzamide and 2-chloro-*N*-(5,5-dimethyl-3-oxo­cyclo­hex-1-en-1-yl)-4-eth­oxy-3,5-di­meth­oxy­benzamide have been determined.

## Chemical context   

Enamino­nes are compounds in which a nitro­gen atom is conjugated through a carbon–carbon double bond to an ester (vinyl­ogous urethane) or a ketone (vinyl­ogous amide) functional group (see Scheme). Enamino­nes may be viewed as amides into which a vinyl fragment has been inter­polated. Designations often used, such as enamino ketone or β-amino-α, β-unsaturated ketone, are misleading in that the compounds rarely exhibit the physical properties normally associated with ketones. Enamino­nes, compounds possessing the structural unit NH_2_—C=C—C=O, are versatile synthetic inter­mediates that combine the ambient nucleophilicity of enamines with the ambient electrophilicity of enones (Greenhill, 1976 ▸; Lue & Greenhill, 1996 ▸).

β-Enamino­nes may be used in the synthesis of many bioactive mol­ecules with a heterocyclic unit. Enamino­nes as inter­mediates are responsible for a wide range of therapeutic agents from both natural and synthetic sources including taxol, anti­convulsants, anti-inflammatories, and duocarmycin, and consequently have been the subject of numerous structural bioactivity investigations in recent times (Misra *et al.*, 2008 ▸; Greenhill, 1977 ▸; Boger *et al.*, 1989 ▸; Eddington *et al.*, 2003 ▸; Stoltz *et al.*, 2016 ▸; Jerach & Elassar, 2015 ▸; Kalita *et al.*, 2017 ▸). In spite of the breadth of research related to the biological properties of enamino­nes, recent research also indicates that enamino­nes, particularly the cyclic 3-(phenyl­amino)-2-cyclo­hexen-1-one (PACO), contain spectroscopic signatures of intra­molecular charge transfer (ICT), making cyclic enamino­nes ideal components for mol­ecules that mimic natural photosynthetic energy and electron transfer (Lue & Greenhill, 1996 ▸). A later study conducted in 2009 concluded that PACO has a low lying strongly polar singlet excited state with significant intra­molecular charge transfer (Misra *et al.*, 2009 ▸).

We herein describe the synthesis and structural characterization of the title benzoyl amides 2-chloro-4-eth­oxy-3,5-dimeth­oxy-*N*-3-oxo­cyclo­hex-1-en-1-yl)benzamide, **3a** and 2-chloro-*N*-(5,5-dimethyl-3-oxo­cyclo­hex-1-en-1-yl)-4-eth­oxy-3,5-di­meth­oxy­benzamide, **3b** developed in connection with an ongoing research inter­est.
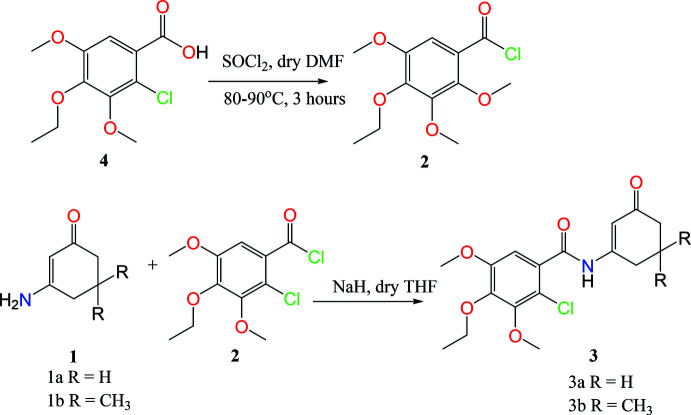


## Structural commentary   

**Figure 1:**
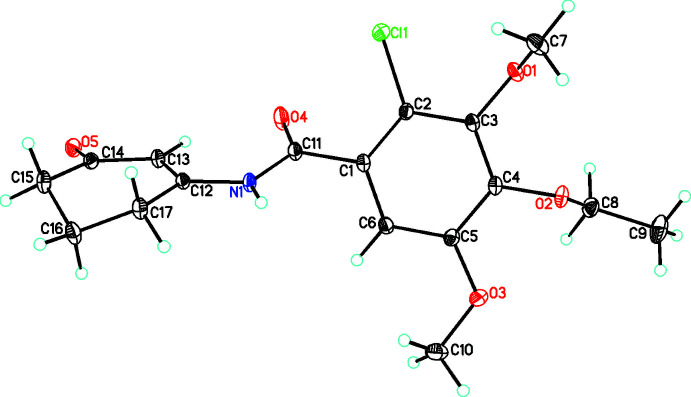
The mol­ecular structure of **3a** with atom labeling and with atomic displacement parameters shown at the 30% probability level.

In view of the bioactivity of enamino­nes, the conformation adopted by a mol­ecule is crucial to its activity. Thus an analysis of this for both mol­ecules is appropriate. The benzoyl amide, C_17_H_20_ClNO_5_ (**3a**), crystallizes in the monoclinic space group *P*2_1_/*c* with *Z* = 4. The compound is the result of the condensation of the enaminone **1a** with the acid chloride **2**. In the case of **3a** (Fig. 1 ▸), the central phenyl ring makes a dihedral angle of 50.8 (3)° with the amide moiety; with the C=O group on the same side of the mol­ecule as the C—Cl group; in the 3-oxo­cyclo­hex-1-en-1-yl group the C=O moiety is on the same side with respect to the phenyl ring [the pseudo torsion angle for O4—C11⋯C14—O5 = 21.8 (1)°]. One of the meth­oxy groups (O3—C10) attached to the C1–C6 benzene ring is close to the plane of the ring [torsion angle between the ring and C5—O3—C10 = 17.72 (2)°], while the eth­oxy and the other meth­oxy substituent are arranged on opposite sides of the ring with the eth­oxy group occupying the same side of the ring as the C=O group in the amide moiety [C8—O2⋯C11—O4 = −44.0 (1) and C7—O1⋯C11—O4 = 123.6 (1)°]. The extended conformation of the eth­oxy group with respect to the ring is shown by a torsion angle of −170.8 (1)° for C4—O2—C8—C9.

The benzoyl amide, C_19_H_24_ClNO_5_ (**3b**), crystallizes in the monoclinic space group *P*2_1_/*c* with *Z* = 8 (*Z*′ = 2), thus there are two independent mol­ecules in the asymmetric unit. The compound is the result of the condensation of the enaminone **1b** with the acid chloride **2**. For one of the two mol­ecules, both the amide and 5,5-dimethyl-3-oxo­cyclo­hex-1-en-1-yl moieties are disordered over two inequivalent conformations with occupancies of 0.551 (2)/0.449 (2). The major difference between the two conformers is due to the conformation adopted by the cyclo­hex-2-en-1-one ring (*vide infra*).

**Figure 2:**
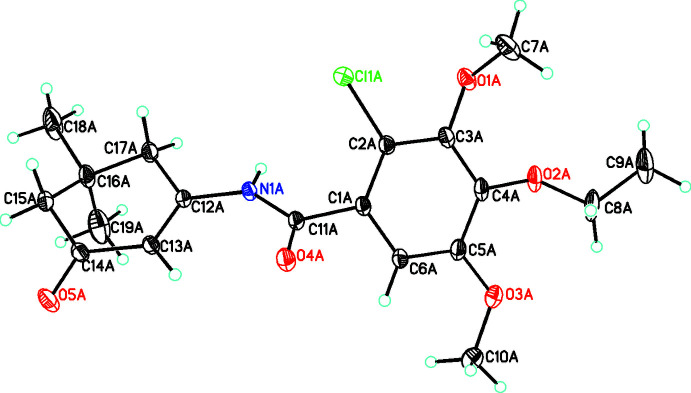
The mol­ecular structure of **3ba** with atom labeling and with atomic displacement parameters shown at the 30% probability level.

The conformations of both independent mol­ecules will be discussed separately and then comparisons will be made between the conformation of **3a** and the two mol­ecules of **3b** in which, due to disorder, one has adopted two different conformations. For simplicity, these will be called **3ba**, **3bb** and **3bc** (where **3bb** and **3bc** are the major and minor components, respectively, of the disordered mol­ecule). For **3ba** (Fig. 2 ▸) the central phenyl ring makes a dihedral angle of 54.5 (3)° with the amide moiety with the C=O group on the opposite side of the mol­ecule as the C—Cl group in contrast to the situation in **3a** (this is illustrated by the respective C2—C1⋯C11—O4 torsion angles of 47.2 (2) and −129.5 (2) for **3a** and **3ba**, respectively). In both the amide moiety and the 3-oxo­cyclo­hex-1-en-1-yl group, the C=O moiety is on the same side [the torsion angle for O4*A*—C11*A*⋯C14*A*—O5*A* = −17.5 (1)°]. For the substituents on the phenyl ring, one meth­oxy group is almost coplanar with the ring [torsion angle between the ring and C5*A*—O3*A*—C10*A* = 3.5 (2)°] while in contrast to the situation in **3a**, both the other meth­oxy and eth­oxy substituents are on the same side of the ring [torsion angles for C7*A*—O1*A*⋯C11*A*—O4*A* and C8*A*—O2*A*⋯C11*A*—O4*A* = −32.4 (2) and −6.4 (2)°, respectively]. The conformation of the eth­oxy substituent is different than that in **3**a in that it has not adopted a fully extended aspect [C4*A*—O2*A*—C8*A*—C9*A* = −148.77 (16)].

**Figure 3:**
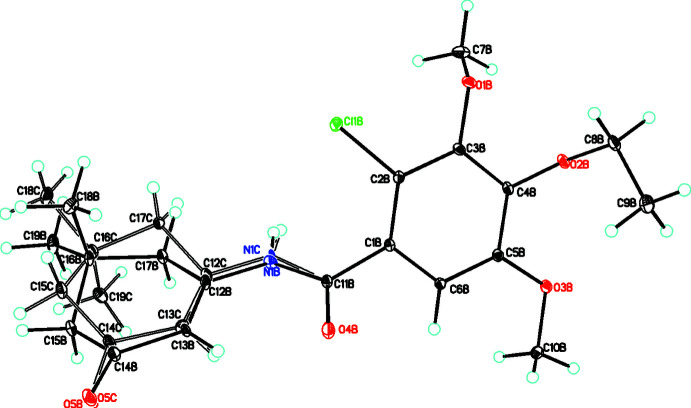
The mol­ecular structure of the disordered mol­ecule in **3b** showing both disorder components (**3bb** and **3bc**) with atom labeling and with atomic displacement parameters shown at the 30% probability level.

As indicated above, **3bb** and **3b**c are the major and minor components of the disordered 5,5-dimethyl-3-oxo­cyclo­hex-1-en-1-yl moieties with occupancies of 0.551 (2)/0.449 (2) (Fig. 3 ▸). The difference in the conformation of this group can be seen by the torsion angles for the C12—C17—C16—C15 grouping in **3a**, **3ba**, **3bb** and **3bc** of −48.67 (17), 50.11 (15), −51.7 (7) and 53.9 (10)°, respectively. From this it can be seen that for this moiety, **3a** and **3bb** have a similar conformation and **3ba** and **3bc** also have a similar conformation. For **3bb**, the central phenyl ring makes a dihedral angle of 55.8 (9)° with the amide moiety with the C=O group on the opposite side of the mol­ecule as the C—Cl group [torsion angle for C2*B*—C1*B*⋯C11*B*—O4*B* = −122.81 (13)]. In both the amide moiety and the 3-oxo­cyclo­hex-1-en-1-yl group, the C=O moiety is on the same side [O4*B*—C11*B*⋯C14*B*—O5*B* = 13.7 (2)°]. For the substituents on the phenyl ring, one meth­oxy group is almost coplanar with the ring [torsion angle between ring and meth­oxy group of 2.3 (2)] while the other meth­oxy group and eth­oxy groups are on opposite sides of the ring [torsion angles for C7*B*—O1*B*⋯C11*B*—O4*B* and C8*B*—O2*B*⋯C11*B*—O4*B* = 165.4 (2) and −46.6 (2)°, respectively]. The conformation of the eth­oxy substituent is different than that in **3a** in that it has not adopted an extended aspect [C4*B*—O2*B*—C8*B*—C9*B* = 67.92 (16)°].

Both **3bb** and **3bc** retain the same (undisordered) phenyl moiety and the only differences are in the conformation of the 5,5-dimethyl-3-oxo­cyclo­hex-1-en-1-yl moiety, thus in discussing this mol­ecule we only have to consider the amide moiety and the 3-oxo­cyclo­hex-1-en-1-yl group where the C=O moiety is on the same side [O4*B*—C11*B*⋯C14*C*—O5*C* = −9.1 (2)°].

## Supra­molecular features   

**Figure 4:**
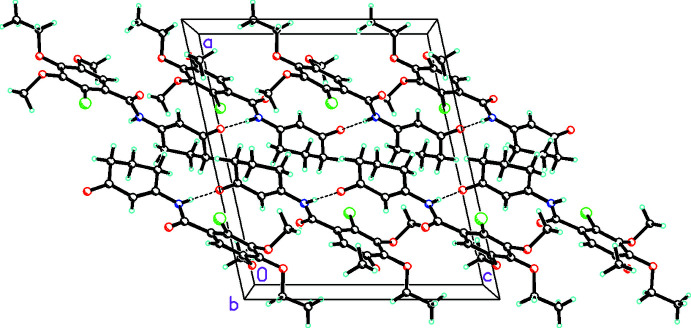
Packing diagram for **3a** viewed along the *b* axis showing the mol­ecules linked by N—H⋯O hydrogen bonds (shown by dashed bonds) into chains propagating in the [001] direction

**Figure 5:**
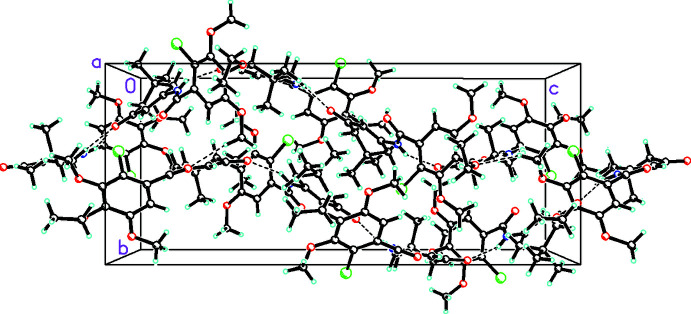
Packing diagram for **3b** viewed along the *a* axis showing the mol­ecules linked by both C—H⋯O and C—H⋯Cl inter­actions as well as N—H⋯O hydrogen bonds (all shown by dashed bonds) into chains propagating in the [001] direction

For **3a**, N—H⋯O hydrogen bonds (Table 1 ▸) link the mol­ecules into a zigzag chain propagating in the [001] direction as shown in Fig. 4 ▸. For **3b**, a combination of C—H⋯O and N—H⋯O inter­molecular inter­actions (Table 2 ▸) link the mol­ecules into a zigzag ribbon propagating in the [001] direction (Fig. 5 ▸).

## Database survey   

A survey of the Cambridge Structural Database for similar compounds did not provide any hits. Even if the mol­ecules are broken up into two components, one based on the tris­ubstituted phenyl ring and the other on the cyclo­hexene ring no hits for the former and only one hit for the latter fragment is obtained [Cambridge Structural Database refcode MOLPUA (Meng *et al.*, 2014 ▸)]. Even in this structure the only similar chromophore is the cyclo­hex-2-ene-1-one fragment, but with the double bond in a different position in the ring. For similar structures to this fragment but containing a cyclo­hexane ring there are DOSDOE, DOSBUK (Romney *et al.*, 2014 ▸) and KAVDAP (Alford *et al.*, 2016 ▸).

## Synthesis and crystallization   

The methodology involves N-deprotonation of the commercially available enamino­nes **1a,b** with sodium hydride followed by benzoyl­ation of **2** to give the title benzoyl amides **3a**,**b** in 54% and 51% yield, respectively, from a method previously reported (see Scheme 1; Anderson *et al.*, 2004 ▸). Benzoyl chloride **2** was prepared *via* chlorination of commercially available **4** under previously reported conditions (Zheng *et al.*, 2011 ▸).

**Preparation of 2-chloro-4-eth­oxy-3,5-di­meth­oxy­benzoyl chloride (2)**

A solution of commercially available 2-chloro-4-eth­oxy-3,5-di­meth­oxy­benzoic acid, **4** (2.07 g, 7.7 mmol), and a catalytic amount of DMF in thionyl chloride (5 ml) was stirred at 353–363 K for 3 h to give the crude acid chloride **2**. The mixture was concentrated under reduced pressure and used without any further purification. ^1^H NMR: (400 MHz, DMSO): δ 1.40–1.45 (3H, *t*, CH_3_), δ 3.03 (3H, *s*, CH_3_), δ 3.19 (3H, *s*, CH_3_), 4.21–4.28 (2H, *q*, CH_2_), 7.48 (H, *s*, aromatic H).

**Preparation of 2-chloro-4-eth­oxy-3,5-dimeth­oxy-** ***N*** **-(3-oxo­cyclo­hex-1-en-1-yl)benzamide (3a)**

The enaminone, **1a** (0.799 g, 7.2 mmol), under an inert atmosphere, was stirred in a solution of NaH (0.391 g, 17.2 mmol) in dry THF (40 ml) maintaining the temperature below 293 K. The reaction was refluxed for 20 minutes, cooled to room temperature and stirred on an ice-bath for 5 minutes before a solution of benzoyl chloride **2** (2.09 g, 7.5 mmol) in dry THF (10 ml) was added dropwise over 5 minutes. After stirring at room temperature for a further 10 minutes, the mixture was quenched with concentrated hydro­chloric acid (∼5 ml) and diluted with di­chloro­methane (25 ml). The mixture was transferred to a separatory funnel and washed successively with water (25 ml), 10% NaHCO_3_ and with water again. The organic layer was dried over sodium sulfate and concentrated *in vacuo*. The crude residue was purified by column chromatography (silica gel, EtOAc:hexa­nes = 5:5) to give compound **3a** (1.37 g, 54%) as a faint yellow solid. (m.p. = 417–418 K) *R*_f_ (EtOAc:hexa­nes 7:3) ^1^H NMR: (400 MHz, DMSO): 1.01 (6H, *s*, 2 × CH_3_), δ 1.27–1.32 (3H, *t*, CH_3_), 2.16 (2H, *m*, CH_2_), 2.43 (2H, *t*, CH_2_), 3.83 (6H, *s*, 2 × CH_3_), δ4.01–4.07 (2H, *quart*, CH_2_), δ 6.70 (H, *s*, CH), 7.04 (H, *s*, aromatic H), 10.25 (H, *s*, NH) ppm; ^13^C NMR (DMSO) δ 198.60, 165.69, 154.02, 152.27, 149.54, 142.88, 131.24, 115.82, 110.15, 107.81, 68.80, 60.83, 56.32, 49.96, 40.85, 32.13, 27.73, 15.35 ppm.

**Preparation of 2-chloro-** ***N*** **-(5,5-dimethyl-3-oxo­cyclo­hex-1-en-1-yl)-4-eth­oxy-3,5-δi­meth­oxy­benzamide (3b)**

The same synthesis and purification method as for **3a** was used to prepare **3b** except that 1.00 g (7.2 mmol) of the enaminone **1b** replaced **1a**: this gave compound **3b** (1.40 g, 51%) as a light white solid. (m.p. = 331–332 K) *R*_f_ (EtOAc:hexa­nes 7:3) ^1^H NMR: (400 MHz, DMSO): δ 1.27–1.32 (3H, *t*, CH_3_), δ 1.8.7–1.95 (2H, quintet, CH_2_), 2.22–2.29 (2H, *t*, CH_2_), 3.32 (6, *s*, 2 × CH_3_, slight long-range coupling noticed), δ 4.00–4.06 (2H, *quart*, CH_2_), δ 6.71 (H, *s*, CH), 7.04 (H, *s*, aromatic H), 10.30 (H, *s*, NH) ppm; ^13^C NMR (DMSO) δ 198.62, 165.56, 156.14, 152.27, 149.55, 142.85, 131.27, 115.77, 110.76, 107.76, 68.80, 60.83, 56.32, 49.96, 40.85, 32.13, 27.73, 21.11, 15.35 ppm.

For both **3a** and **3b** crystals were grown from a 2:1 ethanol:water mixed solvent system.

## Refinement   

Crystal data, data collection and structure refinement details are summarized in Table 3 ▸. The N-bound H atoms were located in difference maps and their positions were freely refined. A riding model was used for the H atoms attached to C with C—H distances ranging from 0.95 to 0.99 Å and *U*_iso_(H) = 1.2*U*_eq_(C) [1.5*U*_eq_(CH_3_)]. For **3b** there are two independent mol­ecules in the asymmetric unit, in one of which the 5,5-dimethyl-3-oxo­cyclo­hex-1-en-1-yl moiety is disordered and was treated with similar metrical parameters with refined occupancies of 0.551 (2)/0.449 (2).

## Supplementary Material

Crystal structure: contains datablock(s) 3a, 3b, global. DOI: 10.1107/S2056989021001778/hb7962sup1.cif

Structure factors: contains datablock(s) 3a. DOI: 10.1107/S2056989021001778/hb79623asup2.hkl

Structure factors: contains datablock(s) 3b. DOI: 10.1107/S2056989021001778/hb79623bsup3.hkl

Supporting information file. DOI: 10.1107/S2056989021001778/hb79623asup4.cml

Supporting information file. DOI: 10.1107/S2056989021001778/hb79623bsup5.cml

CCDC references: 2025600, 2025601

Additional supporting information:  crystallographic information; 3D view; checkCIF report

## Figures and Tables

**Table 1:** Hydrogen-bond geometry (Å, °) for **3a** [Chem scheme1]

*D*—H⋯*A*	*D*—H	H⋯*A*	*D*⋯*A*	*D*—H⋯*A*
N1—H1*A*⋯O5^i^	0.844 (18)	2.098 (18)	2.9410 (15)	177.1 (16)
C7—H7*A*⋯Cl1^ii^	0.98	2.86	3.7022 (16)	145
C7—H7*A*⋯O4^ii^	0.98	2.48	3.293 (2)	140
C9—H9*A*⋯O4^iii^	0.98	2.53	3.470 (2)	161

Symmetry codes: (i) x, -y+{\script{3\over 2}}, z+{\script{1\over 2}}; (ii) x, -y+{\script{1\over 2}}, z+{\script{1\over 2}}; (iii) -x, -y+1, -z+1.

**Table 2:** Hydrogen-bond geometry (Å, °) for **3b** [Chem scheme1]

*D*—H⋯*A*	*D*—H	H⋯*A*	*D*⋯*A*	*D*—H⋯*A*
N1*A*—H1*AA*⋯O5*B*	0.853 (16)	2.040 (18)	2.849 (8)	158.1 (15)
N1*A*—H1*AA*⋯O5*C*	0.853 (16)	2.081 (19)	2.909 (9)	163.5 (15)
C7*A*—H7*AA*⋯O2*B* ^i^	0.98	2.44	3.3451 (17)	153
C8*A*—H8*AB*⋯Cl1*A* ^ii^	0.99	2.92	3.703 (2)	137
C17*A*—H17*A*⋯O5*B*	0.99	2.42	3.285 (7)	146
C17*A*—H17*A*⋯O5*C*	0.99	2.63	3.481 (8)	144
C10*B*—H10*F*⋯O4*A* ^iii^	0.98	2.46	3.4013 (15)	161
N1*B*—H1*BA*⋯O5*A* ^iv^	0.77 (5)	2.31 (5)	2.985 (10)	147 (5)
N1*C*—H1*CA*⋯O5*A* ^iv^	0.88 (5)	1.96 (4)	2.795 (12)	159 (4)
C17*C*—H17*E*⋯O5*A* ^iv^	0.99	2.54	3.364 (3)	141

Symmetry codes: (i) -x+1, y+{\script{1\over 2}}, -z+{\script{1\over 2}}; (ii) -x+2, -y+2, -z+1; (iii) -x+1, -y+2, -z+1; (iv) x, -y+{\script{3\over 2}}, z-{\script{1\over 2}}.

**Table 3:** Experimental details

	**3a**	**3b**
Crystal data		
Chemical formula	C_17_H_20_ClNO_5_	C_19_H_24_ClNO_5_
*M*_r_	353.79	381.84
Crystal system, space group	Monoclinic, *P*2_1_/*c*	Monoclinic, *P*2_1_/*c*
Temperature (K)	150	150
*a*, *b*, *c* (Å)	14.654 (3), 8.9148 (17), 13.045 (2)	14.6986 (13), 10.6309 (10), 25.131 (2)
β (°)	102.581 (3)	90.1851 (14)
*V* (Å^3^)	1663.2 (5)	3926.9 (6)
*Z*	4	8
Radiation type	Mo *K*α	Mo *K*α
μ (mm^−1^)	0.26	0.22
Crystal size (mm)	0.34 × 0.32 × 0.10	0.48 × 0.44 × 0.21

Data collection		
Diffractometer	Bruker SMART APEXII CCD	Bruker SMART APEXII CCD
Absorption correction	Multi-scan (*SADABS*; Sheldrick, 2008 ▸)	Multi-scan (*SADABS*; Sheldrick, 2008 ▸)
*T*_min_, *T*_max_	0.885, 0.975	0.841, 0.954
No. of measured, independent and observed [*I* > 2σ(*I*)] reflections	21434, 5291, 4023	67292, 12630, 10320
*R*_int_	0.041	0.026
(sin θ/λ)_max_ (Å^−1^)	0.726	0.727

Refinement		
*R*[*F* ^2^ > 2σ(*F* ^2^)], *wR*(*F* ^2^), *S*	0.040, 0.111, 1.05	0.039, 0.115, 1.03
No. of reflections	5291	12630
No. of parameters	224	584
No. of restraints	0	399
H-atom treatment	H atoms treated by a mixture of independent and constrained refinement	H atoms treated by a mixture of independent and constrained refinement
Δρ_max_, Δρ_min_ (e Å^−3^)	0.41, −0.25	0.64, −0.35

Computer programs: *APEX2* and *SAINT* (Bruker, 2010 ▸), *SHELXS97* and *SHELXTL* (Sheldrick, 2008 ▸), and *SHELXL2018/3* (Sheldrick, 2015 ▸).

## supplementary crystallographic information

### 2-Chloro-4-ethoxy-3,5-dimethoxy-N-(3-oxocyclohex-1-en-1-yl)benzamide (3a) Crystal data

**Table:**

C_17_H_20_ClNO_5_	*F*(000) = 744
*M**_r_* = 353.79	*D*_x_ = 1.413 Mg m^−^^3^
Monoclinic, *P*2_1_/*c*	Mo *K*α radiation, λ = 0.71073 Å
*a* = 14.654 (3) Å	Cell parameters from 5173 reflections
*b* = 8.9148 (17) Å	θ = 2.7–31.0°
*c* = 13.045 (2) Å	µ = 0.26 mm^−^^1^
β = 102.581 (3)°	*T* = 150 K
*V* = 1663.2 (5) Å^3^	Prism, yellow
*Z* = 4	0.34 × 0.32 × 0.10 mm

### 2-Chloro-4-ethoxy-3,5-dimethoxy-N-(3-oxocyclohex-1-en-1-yl)benzamide (3a) Data collection

**Table:**

Bruker SMART APEXII CCD diffractometer	5291 independent reflections
Radiation source: sealed tube	4023 reflections with *I* > 2σ(*I*)
Detector resolution: 8.333 pixels mm^-1^	*R*_int_ = 0.041
φ and ω scans	θ_max_ = 31.1°, θ_min_ = 2.7°
Absorption correction: multi-scan (SADABS; Sheldrick, 2008)	*h* = −21→21
*T*_min_ = 0.885, *T*_max_ = 0.975	*k* = −12→12
21434 measured reflections	*l* = −18→18

### 2-Chloro-4-ethoxy-3,5-dimethoxy-N-(3-oxocyclohex-1-en-1-yl)benzamide (3a) Refinement

**Table:**

Refinement on *F*^2^	Primary atom site location: structure-invariant direct methods
Least-squares matrix: full	Secondary atom site location: difference Fourier map
*R*[*F*^2^ > 2σ(*F*^2^)] = 0.040	Hydrogen site location: mixed
*wR*(*F*^2^) = 0.111	H atoms treated by a mixture of independent and constrained refinement
*S* = 1.05	*w* = 1/[σ^2^(*F*_o_^2^) + (0.0464*P*)^2^ + 0.4383*P*] where *P* = (*F*_o_^2^ + 2*F*_c_^2^)/3
5291 reflections	(Δ/σ)_max_ < 0.001
224 parameters	Δρ_max_ = 0.41 e Å^−^^3^
0 restraints	Δρ_min_ = −0.25 e Å^−^^3^

### 2-Chloro-4-ethoxy-3,5-dimethoxy-N-(3-oxocyclohex-1-en-1-yl)benzamide (3a) Special details

**Table:**

Geometry. All esds (except the esd in the dihedral angle between two l.s. planes) are estimated using the full covariance matrix. The cell esds are taken into account individually in the estimation of esds in distances, angles and torsion angles; correlations between esds in cell parameters are only used when they are defined by crystal symmetry. An approximate (isotropic) treatment of cell esds is used for estimating esds involving l.s. planes.
Refinement. Compound #4

### 2-Chloro-4-ethoxy-3,5-dimethoxy-N-(3-oxocyclohex-1-en-1-yl)benzamide (3a) Fractional atomic coordinates and isotropic or equivalent isotropic displacement parameters (Å^2^)

**Table:**

	*x*	*y*	*z*	*U*_iso_*/*U*_eq_	
Cl1	0.29234 (3)	0.32109 (4)	0.48615 (3)	0.02725 (10)	
O1	0.18273 (7)	0.38773 (11)	0.64208 (7)	0.0241 (2)	
O2	0.10261 (6)	0.65866 (12)	0.67309 (7)	0.0239 (2)	
O3	0.10408 (7)	0.88836 (11)	0.54071 (9)	0.0302 (2)	
O4	0.25869 (8)	0.47397 (12)	0.27145 (8)	0.0320 (3)	
O5	0.38442 (7)	0.56929 (12)	−0.02661 (7)	0.0249 (2)	
N1	0.33695 (8)	0.69571 (13)	0.31234 (8)	0.0191 (2)	
H1A	0.3526 (12)	0.762 (2)	0.3588 (13)	0.026 (4)*	
C1	0.23398 (9)	0.60558 (15)	0.42223 (10)	0.0193 (2)	
C2	0.23353 (9)	0.48844 (14)	0.49310 (10)	0.0189 (2)	
C3	0.18745 (9)	0.50552 (14)	0.57581 (10)	0.0186 (2)	
C4	0.14420 (9)	0.64091 (15)	0.58915 (10)	0.0194 (2)	
C5	0.14667 (9)	0.76025 (15)	0.51996 (10)	0.0204 (2)	
C6	0.19046 (9)	0.74103 (15)	0.43585 (10)	0.0207 (3)	
H6A	0.190600	0.820783	0.387583	0.025*	
C7	0.25364 (12)	0.39211 (18)	0.73685 (12)	0.0323 (3)	
H7A	0.247923	0.303626	0.779583	0.048*	
H7B	0.246122	0.483071	0.776384	0.048*	
H7C	0.315408	0.392639	0.719441	0.048*	
C8	0.00347 (10)	0.62546 (17)	0.64724 (12)	0.0260 (3)	
H8A	−0.006469	0.516730	0.633878	0.031*	
H8B	−0.027186	0.680643	0.583095	0.031*	
C9	−0.03754 (11)	0.6722 (2)	0.73783 (14)	0.0410 (4)	
H9A	−0.104576	0.649464	0.722087	0.061*	
H9B	−0.028357	0.780238	0.749697	0.061*	
H9C	−0.006531	0.617541	0.801030	0.061*	
C10	0.12623 (12)	1.02402 (17)	0.49314 (14)	0.0327 (3)	
H10A	0.109255	1.110196	0.531760	0.049*	
H10B	0.091199	1.028074	0.420061	0.049*	
H10C	0.193419	1.026858	0.494974	0.049*	
C11	0.27614 (9)	0.58307 (16)	0.32791 (10)	0.0213 (3)	
C12	0.38339 (9)	0.70472 (14)	0.22986 (10)	0.0180 (2)	
C13	0.36265 (9)	0.61821 (15)	0.14259 (10)	0.0204 (3)	
H13A	0.315554	0.543565	0.137043	0.024*	
C14	0.41092 (9)	0.63713 (15)	0.05714 (10)	0.0201 (2)	
C15	0.49279 (10)	0.74267 (18)	0.07335 (11)	0.0262 (3)	
H15A	0.499687	0.781566	0.004357	0.031*	
H15B	0.550511	0.686924	0.104906	0.031*	
C16	0.48180 (11)	0.87402 (17)	0.14418 (11)	0.0275 (3)	
H16A	0.430304	0.939526	0.107746	0.033*	
H16B	0.540060	0.934001	0.158934	0.033*	
C17	0.46077 (10)	0.81930 (16)	0.24729 (11)	0.0234 (3)	
H17A	0.518006	0.774546	0.291063	0.028*	
H17B	0.442654	0.905888	0.286032	0.028*	

### 2-Chloro-4-ethoxy-3,5-dimethoxy-N-(3-oxocyclohex-1-en-1-yl)benzamide (3a) Atomic displacement parameters (Å^2^)

**Table:**

	*U*^11^	*U*^22^	*U*^33^	*U*^12^	*U*^13^	*U*^23^
Cl1	0.03470 (19)	0.02092 (16)	0.02974 (18)	0.00364 (13)	0.01494 (14)	−0.00058 (13)
O1	0.0298 (5)	0.0226 (5)	0.0213 (5)	−0.0047 (4)	0.0089 (4)	0.0046 (4)
O2	0.0204 (5)	0.0342 (5)	0.0200 (5)	−0.0020 (4)	0.0107 (4)	−0.0029 (4)
O3	0.0330 (6)	0.0225 (5)	0.0407 (6)	0.0067 (4)	0.0200 (5)	0.0031 (4)
O4	0.0448 (6)	0.0290 (5)	0.0281 (5)	−0.0144 (5)	0.0210 (5)	−0.0079 (4)
O5	0.0320 (5)	0.0273 (5)	0.0173 (4)	0.0001 (4)	0.0092 (4)	−0.0014 (4)
N1	0.0220 (5)	0.0222 (5)	0.0153 (5)	−0.0033 (4)	0.0087 (4)	−0.0023 (4)
C1	0.0192 (6)	0.0227 (6)	0.0177 (6)	−0.0027 (5)	0.0076 (4)	0.0000 (5)
C2	0.0194 (6)	0.0191 (6)	0.0202 (6)	−0.0008 (5)	0.0085 (4)	−0.0009 (5)
C3	0.0189 (6)	0.0203 (6)	0.0176 (6)	−0.0035 (5)	0.0065 (4)	0.0012 (5)
C4	0.0187 (6)	0.0239 (6)	0.0173 (6)	−0.0023 (5)	0.0075 (4)	−0.0007 (5)
C5	0.0181 (6)	0.0215 (6)	0.0227 (6)	0.0005 (5)	0.0071 (5)	0.0002 (5)
C6	0.0205 (6)	0.0222 (6)	0.0206 (6)	0.0003 (5)	0.0072 (5)	0.0039 (5)
C7	0.0401 (9)	0.0305 (8)	0.0242 (7)	−0.0003 (6)	0.0026 (6)	0.0077 (6)
C8	0.0210 (6)	0.0297 (7)	0.0301 (7)	−0.0005 (5)	0.0114 (5)	−0.0007 (6)
C9	0.0256 (8)	0.0645 (12)	0.0379 (9)	0.0013 (8)	0.0178 (7)	−0.0059 (8)
C10	0.0356 (8)	0.0216 (7)	0.0420 (9)	0.0061 (6)	0.0107 (7)	0.0059 (6)
C11	0.0230 (6)	0.0242 (6)	0.0190 (6)	−0.0021 (5)	0.0094 (5)	0.0011 (5)
C12	0.0183 (6)	0.0206 (6)	0.0165 (5)	0.0011 (4)	0.0065 (4)	0.0028 (4)
C13	0.0217 (6)	0.0245 (6)	0.0165 (6)	−0.0025 (5)	0.0072 (4)	0.0009 (5)
C14	0.0220 (6)	0.0229 (6)	0.0165 (6)	0.0032 (5)	0.0065 (4)	0.0027 (5)
C15	0.0258 (7)	0.0357 (8)	0.0202 (6)	−0.0046 (6)	0.0116 (5)	−0.0005 (6)
C16	0.0305 (7)	0.0290 (7)	0.0263 (7)	−0.0101 (6)	0.0132 (6)	−0.0001 (6)
C17	0.0240 (6)	0.0274 (7)	0.0208 (6)	−0.0066 (5)	0.0096 (5)	−0.0030 (5)

### 2-Chloro-4-ethoxy-3,5-dimethoxy-N-(3-oxocyclohex-1-en-1-yl)benzamide (3a) Geometric parameters (Å, º)

**Table:**

Cl1—C2	1.7352 (13)	C8—C9	1.497 (2)
O1—C3	1.3714 (15)	C8—H8A	0.9900
O1—C7	1.4317 (18)	C8—H8B	0.9900
O2—C4	1.3734 (14)	C9—H9A	0.9800
O2—C8	1.4486 (16)	C9—H9B	0.9800
O3—C5	1.3567 (16)	C9—H9C	0.9800
O3—C10	1.4285 (18)	C10—H10A	0.9800
O4—C11	1.2132 (17)	C10—H10B	0.9800
O5—C14	1.2350 (16)	C10—H10C	0.9800
N1—C11	1.3865 (17)	C12—C13	1.3536 (18)
N1—C12	1.3950 (15)	C12—C17	1.5061 (18)
N1—H1A	0.844 (18)	C13—C14	1.4543 (17)
C1—C6	1.3949 (18)	C13—H13A	0.9500
C1—C2	1.3956 (18)	C14—C15	1.5029 (19)
C1—C11	1.5056 (17)	C15—C16	1.522 (2)
C2—C3	1.4001 (16)	C15—H15A	0.9900
C3—C4	1.3917 (18)	C15—H15B	0.9900
C4—C5	1.4008 (19)	C16—C17	1.5241 (19)
C5—C6	1.3969 (17)	C16—H16A	0.9900
C6—H6A	0.9500	C16—H16B	0.9900
C7—H7A	0.9800	C17—H17A	0.9900
C7—H7B	0.9800	C17—H17B	0.9900
C7—H7C	0.9800		
			
C3—O1—C7	113.37 (11)	C8—C9—H9C	109.5
C4—O2—C8	112.80 (10)	H9A—C9—H9C	109.5
C5—O3—C10	117.91 (11)	H9B—C9—H9C	109.5
C11—N1—C12	126.28 (11)	O3—C10—H10A	109.5
C11—N1—H1A	119.2 (12)	O3—C10—H10B	109.5
C12—N1—H1A	114.3 (12)	H10A—C10—H10B	109.5
C6—C1—C2	119.68 (11)	O3—C10—H10C	109.5
C6—C1—C11	119.99 (11)	H10A—C10—H10C	109.5
C2—C1—C11	120.24 (12)	H10B—C10—H10C	109.5
C1—C2—C3	120.11 (12)	O4—C11—N1	123.29 (11)
C1—C2—Cl1	122.32 (9)	O4—C11—C1	122.22 (12)
C3—C2—Cl1	117.53 (10)	N1—C11—C1	114.48 (11)
O1—C3—C4	119.88 (11)	C13—C12—N1	123.91 (12)
O1—C3—C2	120.10 (11)	C13—C12—C17	122.49 (11)
C4—C3—C2	119.99 (11)	N1—C12—C17	113.59 (11)
O2—C4—C3	119.54 (11)	C12—C13—C14	121.39 (12)
O2—C4—C5	120.30 (12)	C12—C13—H13A	119.3
C3—C4—C5	120.11 (11)	C14—C13—H13A	119.3
O3—C5—C6	124.74 (12)	O5—C14—C13	120.63 (12)
O3—C5—C4	115.67 (11)	O5—C14—C15	121.24 (11)
C6—C5—C4	119.59 (12)	C13—C14—C15	118.12 (11)
C1—C6—C5	120.47 (12)	C14—C15—C16	112.36 (11)
C1—C6—H6A	119.8	C14—C15—H15A	109.1
C5—C6—H6A	119.8	C16—C15—H15A	109.1
O1—C7—H7A	109.5	C14—C15—H15B	109.1
O1—C7—H7B	109.5	C16—C15—H15B	109.1
H7A—C7—H7B	109.5	H15A—C15—H15B	107.9
O1—C7—H7C	109.5	C15—C16—C17	110.98 (12)
H7A—C7—H7C	109.5	C15—C16—H16A	109.4
H7B—C7—H7C	109.5	C17—C16—H16A	109.4
O2—C8—C9	108.30 (12)	C15—C16—H16B	109.4
O2—C8—H8A	110.0	C17—C16—H16B	109.4
C9—C8—H8A	110.0	H16A—C16—H16B	108.0
O2—C8—H8B	110.0	C12—C17—C16	111.97 (11)
C9—C8—H8B	110.0	C12—C17—H17A	109.2
H8A—C8—H8B	108.4	C16—C17—H17A	109.2
C8—C9—H9A	109.5	C12—C17—H17B	109.2
C8—C9—H9B	109.5	C16—C17—H17B	109.2
H9A—C9—H9B	109.5	H17A—C17—H17B	107.9
			
C6—C1—C2—C3	1.71 (19)	C11—C1—C6—C5	176.51 (12)
C11—C1—C2—C3	−174.66 (12)	O3—C5—C6—C1	178.77 (13)
C6—C1—C2—Cl1	−175.87 (10)	C4—C5—C6—C1	−1.9 (2)
C11—C1—C2—Cl1	7.76 (18)	C4—O2—C8—C9	−170.82 (13)
C7—O1—C3—C4	−85.34 (15)	C12—N1—C11—O4	2.7 (2)
C7—O1—C3—C2	96.38 (15)	C12—N1—C11—C1	−178.18 (12)
C1—C2—C3—O1	176.51 (12)	C6—C1—C11—O4	−129.20 (15)
Cl1—C2—C3—O1	−5.80 (17)	C2—C1—C11—O4	47.2 (2)
C1—C2—C3—C4	−1.77 (19)	C6—C1—C11—N1	51.64 (17)
Cl1—C2—C3—C4	175.92 (10)	C2—C1—C11—N1	−132.00 (13)
C8—O2—C4—C3	−94.55 (14)	C11—N1—C12—C13	12.4 (2)
C8—O2—C4—C5	88.21 (15)	C11—N1—C12—C17	−166.70 (13)
O1—C3—C4—O2	4.46 (18)	N1—C12—C13—C14	177.36 (12)
C2—C3—C4—O2	−177.25 (11)	C17—C12—C13—C14	−3.6 (2)
O1—C3—C4—C5	−178.29 (12)	C12—C13—C14—O5	−171.94 (13)
C2—C3—C4—C5	−0.01 (19)	C12—C13—C14—C15	7.3 (2)
C10—O3—C5—C6	−18.0 (2)	O5—C14—C15—C16	146.86 (13)
C10—O3—C5—C4	162.68 (13)	C13—C14—C15—C16	−32.36 (18)
O2—C4—C5—O3	−1.55 (19)	C14—C15—C16—C17	52.88 (17)
C3—C4—C5—O3	−178.78 (12)	C13—C12—C17—C16	24.94 (19)
O2—C4—C5—C6	179.06 (12)	N1—C12—C17—C16	−155.90 (12)
C3—C4—C5—C6	1.8 (2)	C15—C16—C17—C12	−48.67 (17)
C2—C1—C6—C5	0.1 (2)		

### 2-Chloro-4-ethoxy-3,5-dimethoxy-N-(3-oxocyclohex-1-en-1-yl)benzamide (3a) Hydrogen-bond geometry (Å, º)

**Table:**

*D*—H···*A*	*D*—H	H···*A*	*D*···*A*	*D*—H···*A*
N1—H1*A*···O5^i^	0.844 (18)	2.098 (18)	2.9410 (15)	177.1 (16)
C7—H7*A*···Cl1^ii^	0.98	2.86	3.7022 (16)	145
C7—H7*A*···O4^ii^	0.98	2.48	3.293 (2)	140
C9—H9*A*···O4^iii^	0.98	2.53	3.470 (2)	161

Symmetry codes: (i) *x*, −*y*+3/2, *z*+1/2; (ii) *x*, −*y*+1/2, *z*+1/2; (iii) −*x*, −*y*+1, −*z*+1.

### 2-Chloro-N-(5,5-dimethyl-3-oxocyclohex-1-en-1-yl)-4-ethoxy-3,5-dimethoxybenzamide (3b) Crystal data

**Table:**

C_19_H_24_ClNO_5_	*F*(000) = 1616
*M**_r_* = 381.84	*D*_x_ = 1.292 Mg m^−^^3^
Monoclinic, *P*2_1_/*c*	Mo *K*α radiation, λ = 0.71073 Å
*a* = 14.6986 (13) Å	Cell parameters from 23388 reflections
*b* = 10.6309 (10) Å	θ = 2.4–31.0°
*c* = 25.131 (2) Å	µ = 0.22 mm^−^^1^
β = 90.1851 (14)°	*T* = 150 K
*V* = 3926.9 (6) Å^3^	Prism, colourless
*Z* = 8	0.48 × 0.44 × 0.21 mm

### 2-Chloro-N-(5,5-dimethyl-3-oxocyclohex-1-en-1-yl)-4-ethoxy-3,5-dimethoxybenzamide (3b) Data collection

**Table:**

Bruker SMART APEXII CCD diffractometer	12630 independent reflections
Radiation source: sealed tube	10320 reflections with *I* > 2σ(*I*)
Detector resolution: 8.333 pixels mm^-1^	*R*_int_ = 0.026
φ and ω scans	θ_max_ = 31.1°, θ_min_ = 2.1°
Absorption correction: multi-scan (SADABS; Sheldrick, 2008)	*h* = −21→21
*T*_min_ = 0.841, *T*_max_ = 0.954	*k* = −15→15
67292 measured reflections	*l* = −36→36

### 2-Chloro-N-(5,5-dimethyl-3-oxocyclohex-1-en-1-yl)-4-ethoxy-3,5-dimethoxybenzamide (3b) Refinement

**Table:**

Refinement on *F*^2^	Primary atom site location: structure-invariant direct methods
Least-squares matrix: full	Secondary atom site location: difference Fourier map
*R*[*F*^2^ > 2σ(*F*^2^)] = 0.039	Hydrogen site location: mixed
*wR*(*F*^2^) = 0.115	H atoms treated by a mixture of independent and constrained refinement
*S* = 1.03	*w* = 1/[σ^2^(*F*_o_^2^) + (0.0599*P*)^2^ + 1.0458*P*] where *P* = (*F*_o_^2^ + 2*F*_c_^2^)/3
12630 reflections	(Δ/σ)_max_ = 0.006
584 parameters	Δρ_max_ = 0.64 e Å^−^^3^
399 restraints	Δρ_min_ = −0.35 e Å^−^^3^

### 2-Chloro-N-(5,5-dimethyl-3-oxocyclohex-1-en-1-yl)-4-ethoxy-3,5-dimethoxybenzamide (3b) Special details

**Table:**

Geometry. All esds (except the esd in the dihedral angle between two l.s. planes) are estimated using the full covariance matrix. The cell esds are taken into account individually in the estimation of esds in distances, angles and torsion angles; correlations between esds in cell parameters are only used when they are defined by crystal symmetry. An approximate (isotropic) treatment of cell esds is used for estimating esds involving l.s. planes.
Refinement. Compound #6

### 2-Chloro-N-(5,5-dimethyl-3-oxocyclohex-1-en-1-yl)-4-ethoxy-3,5-dimethoxybenzamide (3b) Fractional atomic coordinates and isotropic or equivalent isotropic displacement parameters (Å^2^)

**Table:**

	*x*	*y*	*z*	*U*_iso_*/*U*_eq_	Occ. (<1)
Cl1A	0.74167 (2)	1.06636 (3)	0.50622 (2)	0.03341 (7)	
O1A	0.85300 (6)	0.92470 (9)	0.43031 (3)	0.03358 (19)	
O2A	0.96808 (6)	0.73233 (10)	0.45218 (4)	0.0371 (2)	
O3A	0.98422 (6)	0.64044 (10)	0.55396 (4)	0.0411 (2)	
O4A	0.79762 (5)	0.97694 (9)	0.66166 (3)	0.03064 (18)	
O5A	0.54514 (6)	1.01715 (10)	0.78213 (3)	0.0376 (2)	
N1A	0.67015 (6)	0.94345 (9)	0.61067 (4)	0.02155 (17)	
H1AA	0.6506 (11)	0.9153 (15)	0.5810 (7)	0.034 (4)*	
C1A	0.81762 (7)	0.89610 (10)	0.57423 (4)	0.02277 (19)	
C2A	0.81118 (7)	0.94022 (11)	0.52235 (4)	0.0245 (2)	
C3A	0.86370 (7)	0.88575 (12)	0.48185 (4)	0.0272 (2)	
C4A	0.92222 (7)	0.78611 (12)	0.49363 (5)	0.0293 (2)	
C5A	0.92801 (7)	0.74122 (12)	0.54613 (5)	0.0293 (2)	
C6A	0.87719 (7)	0.79758 (11)	0.58605 (4)	0.0263 (2)	
H6AA	0.882994	0.768878	0.621695	0.032*	
C7A	0.91558 (11)	1.01997 (17)	0.41412 (5)	0.0474 (4)	
H7AA	0.903236	1.043380	0.377078	0.071*	
H7AB	0.977879	0.987895	0.417198	0.071*	
H7AC	0.908551	1.093994	0.436981	0.071*	
C8A	1.06559 (9)	0.7271 (2)	0.45645 (6)	0.0624 (5)	
H8AA	1.083977	0.647258	0.473668	0.075*	
H8AB	1.087544	0.797509	0.478864	0.075*	
C9A	1.10703 (11)	0.7356 (2)	0.40297 (7)	0.0628 (5)	
H9AA	1.172751	0.721418	0.405797	0.094*	
H9AB	1.095707	0.819284	0.388025	0.094*	
H9AC	1.080069	0.671610	0.379685	0.094*	
C10A	0.99347 (11)	0.59578 (18)	0.60744 (7)	0.0550 (4)	
H10A	1.033598	0.522173	0.607983	0.082*	
H10B	0.933524	0.572283	0.621209	0.082*	
H10C	1.019610	0.662303	0.629735	0.082*	
C11A	0.76218 (7)	0.94515 (10)	0.61997 (4)	0.02202 (19)	
C12A	0.60074 (6)	0.97178 (10)	0.64589 (4)	0.02102 (18)	
C13A	0.61233 (7)	0.98962 (10)	0.69867 (4)	0.02312 (19)	
H13A	0.671961	0.987378	0.713341	0.028*	
C14A	0.53549 (7)	1.01213 (11)	0.73334 (4)	0.0258 (2)	
C15A	0.44417 (8)	1.03239 (13)	0.70805 (5)	0.0322 (2)	
H15A	0.437492	1.122477	0.698850	0.039*	
H15C	0.396169	1.011049	0.734077	0.039*	
C16A	0.43022 (7)	0.95307 (15)	0.65763 (5)	0.0363 (3)	
C17A	0.50923 (7)	0.98156 (14)	0.61962 (4)	0.0327 (3)	
H17A	0.506711	0.922034	0.589352	0.039*	
H17B	0.501634	1.067588	0.605191	0.039*	
C18A	0.34099 (9)	0.9921 (3)	0.63114 (6)	0.0782 (8)	
H18A	0.290687	0.979665	0.656099	0.117*	
H18B	0.330783	0.940598	0.599323	0.117*	
H18C	0.344203	1.080952	0.620991	0.117*	
C19A	0.42926 (11)	0.81411 (18)	0.67166 (7)	0.0577 (5)	
H19A	0.380973	0.797814	0.697561	0.087*	
H19B	0.488156	0.790343	0.687029	0.087*	
H19C	0.418020	0.764497	0.639432	0.087*	
Cl1B	0.33068 (2)	0.36791 (3)	0.37042 (2)	0.03251 (7)	
O1B	0.19443 (6)	0.32113 (8)	0.28774 (3)	0.03058 (17)	
O2B	0.12498 (6)	0.49472 (9)	0.21650 (3)	0.03228 (18)	
O3B	0.17637 (6)	0.73524 (8)	0.22015 (3)	0.03071 (18)	
O4B	0.35516 (6)	0.74079 (10)	0.40209 (4)	0.0415 (2)	
C1B	0.31411 (6)	0.60638 (10)	0.33084 (4)	0.02011 (18)	
C2B	0.28768 (7)	0.48116 (10)	0.32756 (4)	0.02208 (19)	
C3B	0.22268 (7)	0.44399 (10)	0.28987 (4)	0.02362 (19)	
C4B	0.18562 (7)	0.53194 (11)	0.25503 (4)	0.0240 (2)	
C5B	0.21397 (7)	0.65796 (10)	0.25735 (4)	0.02301 (19)	
C6B	0.27647 (7)	0.69485 (10)	0.29595 (4)	0.02189 (18)	
H6BA	0.293725	0.780733	0.298645	0.026*	
C7B	0.23634 (11)	0.25212 (13)	0.24515 (7)	0.0436 (3)	
H7BA	0.215324	0.164658	0.245936	0.065*	
H7BB	0.302605	0.254294	0.249483	0.065*	
H7BC	0.219649	0.290379	0.211010	0.065*	
C8B	0.03306 (9)	0.47519 (14)	0.23569 (6)	0.0416 (3)	
H8BA	0.034665	0.416637	0.266315	0.050*	
H8BB	−0.003821	0.435696	0.207205	0.050*	
C9B	−0.01135 (10)	0.59598 (18)	0.25249 (10)	0.0675 (6)	
H9BA	−0.072982	0.578521	0.265278	0.101*	
H9BB	−0.014447	0.653490	0.222061	0.101*	
H9BC	0.024414	0.634758	0.281091	0.101*	
C10B	0.20442 (9)	0.86380 (11)	0.22096 (5)	0.0310 (2)	
H10D	0.173044	0.909934	0.192567	0.047*	
H10E	0.270299	0.868615	0.215424	0.047*	
H10F	0.189185	0.901108	0.255464	0.047*	
C11B	0.37827 (7)	0.65588 (10)	0.37276 (4)	0.02269 (19)	
O5B	0.5926 (5)	0.7923 (8)	0.5286 (3)	0.0383 (12)	0.551 (2)
N1B	0.4614 (6)	0.6071 (10)	0.3766 (4)	0.0274 (15)	0.551 (2)
H1BA	0.471 (3)	0.552 (4)	0.3576 (18)	0.026 (13)*	0.551 (2)
C12B	0.5307 (6)	0.6264 (11)	0.4131 (5)	0.0259 (10)	0.551 (2)
C13B	0.5224 (5)	0.7057 (8)	0.4537 (3)	0.0210 (9)	0.551 (2)
H13B	0.466093	0.746700	0.460177	0.025*	0.551 (2)
C14B	0.6004 (6)	0.7288 (8)	0.4881 (4)	0.0293 (11)	0.551 (2)
C15B	0.69268 (14)	0.6766 (2)	0.47298 (9)	0.0304 (5)	0.551 (2)
H15B	0.729500	0.665206	0.505684	0.036*	0.551 (2)
H15D	0.724367	0.738554	0.450190	0.036*	0.551 (2)
C16B	0.6870 (4)	0.5507 (7)	0.4434 (3)	0.0262 (9)	0.551 (2)
C17B	0.62225 (13)	0.5682 (2)	0.39563 (8)	0.0261 (4)	0.551 (2)
H17C	0.610886	0.485622	0.378636	0.031*	0.551 (2)
H17D	0.651346	0.623624	0.368993	0.031*	0.551 (2)
C18B	0.65099 (17)	0.4478 (2)	0.48043 (10)	0.0384 (5)	0.551 (2)
H18D	0.692949	0.437144	0.510480	0.058*	0.551 (2)
H18E	0.646148	0.368375	0.460797	0.058*	0.551 (2)
H18F	0.590875	0.471931	0.493723	0.058*	0.551 (2)
C19B	0.78134 (15)	0.5136 (3)	0.42324 (10)	0.0436 (7)	0.551 (2)
H19D	0.823551	0.507798	0.453410	0.065*	0.551 (2)
H19E	0.803155	0.577384	0.398177	0.065*	0.551 (2)
H19F	0.777791	0.431964	0.405263	0.065*	0.551 (2)
O5C	0.6059 (7)	0.7977 (10)	0.5201 (4)	0.0413 (15)	0.449 (2)
N1C	0.4643 (5)	0.5949 (11)	0.3708 (4)	0.0156 (9)	0.449 (2)
H1CA	0.476 (3)	0.549 (4)	0.3423 (18)	0.019 (12)*	0.449 (2)
C12C	0.5324 (8)	0.6138 (14)	0.4091 (6)	0.0262 (12)	0.449 (2)
C13C	0.5340 (6)	0.6981 (11)	0.4488 (4)	0.0268 (13)	0.449 (2)
H13C	0.485165	0.756476	0.450818	0.032*	0.449 (2)
C14C	0.6048 (7)	0.7067 (10)	0.4889 (5)	0.0310 (13)	0.449 (2)
C15C	0.67207 (18)	0.6008 (3)	0.49113 (11)	0.0341 (6)	0.449 (2)
H15E	0.649682	0.536354	0.516327	0.041*	0.449 (2)
H15F	0.730553	0.633284	0.505144	0.041*	0.449 (2)
C16C	0.6890 (6)	0.5385 (9)	0.4371 (4)	0.0293 (12)	0.449 (2)
C17C	0.59763 (16)	0.5023 (2)	0.41227 (10)	0.0244 (5)	0.449 (2)
H17E	0.607993	0.468961	0.376009	0.029*	0.449 (2)
H17F	0.569501	0.434700	0.433730	0.029*	0.449 (2)
C18C	0.7464 (2)	0.4183 (3)	0.44515 (14)	0.0434 (8)	0.449 (2)
H18G	0.806403	0.441080	0.459204	0.065*	0.449 (2)
H18H	0.753484	0.375020	0.410976	0.065*	0.449 (2)
H18I	0.715614	0.362431	0.470371	0.065*	0.449 (2)
C19C	0.74033 (19)	0.6285 (3)	0.39994 (12)	0.0373 (7)	0.449 (2)
H19G	0.706920	0.708007	0.397146	0.056*	0.449 (2)
H19H	0.745573	0.590307	0.364584	0.056*	0.449 (2)
H19I	0.801241	0.644531	0.414378	0.056*	0.449 (2)

### 2-Chloro-N-(5,5-dimethyl-3-oxocyclohex-1-en-1-yl)-4-ethoxy-3,5-dimethoxybenzamide (3b) Atomic displacement parameters (Å^2^)

**Table:**

	*U*^11^	*U*^22^	*U*^33^	*U*^12^	*U*^13^	*U*^23^
Cl1A	0.03295 (13)	0.04046 (16)	0.02684 (13)	0.00661 (11)	0.00220 (10)	0.00619 (11)
O1A	0.0289 (4)	0.0525 (5)	0.0193 (4)	−0.0066 (4)	0.0016 (3)	−0.0010 (4)
O2A	0.0223 (4)	0.0560 (6)	0.0330 (4)	−0.0005 (4)	0.0088 (3)	−0.0150 (4)
O3A	0.0336 (4)	0.0458 (5)	0.0439 (5)	0.0154 (4)	0.0105 (4)	0.0032 (4)
O4A	0.0233 (3)	0.0448 (5)	0.0238 (4)	−0.0036 (3)	0.0004 (3)	−0.0060 (3)
O5A	0.0335 (4)	0.0616 (6)	0.0178 (4)	−0.0051 (4)	0.0043 (3)	0.0003 (4)
N1A	0.0193 (4)	0.0277 (4)	0.0176 (4)	−0.0009 (3)	0.0024 (3)	−0.0040 (3)
C1A	0.0181 (4)	0.0292 (5)	0.0211 (4)	−0.0022 (4)	0.0033 (3)	−0.0018 (4)
C2A	0.0195 (4)	0.0320 (5)	0.0220 (5)	−0.0011 (4)	0.0017 (3)	0.0000 (4)
C3A	0.0208 (4)	0.0409 (6)	0.0200 (5)	−0.0046 (4)	0.0029 (4)	−0.0032 (4)
C4A	0.0189 (4)	0.0416 (6)	0.0274 (5)	−0.0031 (4)	0.0070 (4)	−0.0080 (5)
C5A	0.0200 (4)	0.0353 (6)	0.0326 (6)	0.0019 (4)	0.0053 (4)	−0.0016 (5)
C6A	0.0196 (4)	0.0340 (6)	0.0252 (5)	0.0002 (4)	0.0042 (4)	0.0016 (4)
C7A	0.0488 (8)	0.0673 (10)	0.0262 (6)	−0.0183 (7)	0.0043 (5)	0.0060 (6)
C8A	0.0219 (6)	0.1254 (17)	0.0400 (8)	0.0095 (8)	0.0090 (5)	−0.0176 (9)
C9A	0.0377 (7)	0.0939 (14)	0.0570 (10)	−0.0074 (8)	0.0225 (7)	−0.0248 (10)
C10A	0.0494 (8)	0.0604 (10)	0.0552 (9)	0.0266 (8)	0.0120 (7)	0.0177 (8)
C11A	0.0197 (4)	0.0250 (5)	0.0213 (4)	−0.0013 (3)	0.0031 (3)	−0.0002 (4)
C12A	0.0201 (4)	0.0228 (5)	0.0202 (4)	0.0008 (3)	0.0031 (3)	−0.0002 (4)
C13A	0.0214 (4)	0.0287 (5)	0.0193 (4)	−0.0013 (4)	0.0018 (3)	0.0004 (4)
C14A	0.0266 (5)	0.0311 (5)	0.0199 (5)	−0.0002 (4)	0.0039 (4)	0.0008 (4)
C15A	0.0273 (5)	0.0462 (7)	0.0232 (5)	0.0103 (5)	0.0048 (4)	−0.0002 (5)
C16A	0.0187 (4)	0.0670 (9)	0.0232 (5)	0.0029 (5)	0.0020 (4)	−0.0056 (5)
C17A	0.0219 (5)	0.0560 (8)	0.0201 (5)	0.0068 (5)	0.0006 (4)	−0.0016 (5)
C18A	0.0225 (6)	0.180 (2)	0.0325 (7)	0.0184 (10)	−0.0011 (5)	−0.0117 (11)
C19A	0.0454 (8)	0.0640 (11)	0.0639 (10)	−0.0278 (7)	0.0217 (7)	−0.0218 (8)
Cl1B	0.03134 (13)	0.03067 (14)	0.03546 (15)	−0.00338 (10)	−0.00739 (11)	0.01257 (11)
O1B	0.0322 (4)	0.0260 (4)	0.0335 (4)	−0.0104 (3)	−0.0015 (3)	0.0034 (3)
O2B	0.0319 (4)	0.0379 (5)	0.0270 (4)	−0.0106 (3)	−0.0110 (3)	0.0017 (3)
O3B	0.0340 (4)	0.0294 (4)	0.0287 (4)	−0.0042 (3)	−0.0122 (3)	0.0075 (3)
O4B	0.0345 (4)	0.0486 (6)	0.0412 (5)	0.0168 (4)	−0.0129 (4)	−0.0232 (4)
C1B	0.0169 (4)	0.0262 (5)	0.0172 (4)	0.0002 (3)	0.0008 (3)	−0.0015 (4)
C2B	0.0195 (4)	0.0258 (5)	0.0209 (4)	−0.0007 (3)	0.0004 (3)	0.0043 (4)
C3B	0.0222 (4)	0.0248 (5)	0.0239 (5)	−0.0057 (4)	0.0008 (4)	0.0015 (4)
C4B	0.0214 (4)	0.0296 (5)	0.0209 (5)	−0.0053 (4)	−0.0030 (3)	0.0004 (4)
C5B	0.0223 (4)	0.0262 (5)	0.0205 (4)	−0.0012 (4)	−0.0023 (3)	0.0029 (4)
C6B	0.0211 (4)	0.0229 (5)	0.0217 (4)	−0.0012 (3)	−0.0013 (3)	−0.0001 (4)
C7B	0.0510 (8)	0.0262 (6)	0.0538 (9)	−0.0048 (5)	0.0071 (6)	−0.0044 (6)
C8B	0.0284 (6)	0.0426 (7)	0.0536 (8)	−0.0126 (5)	−0.0136 (5)	0.0074 (6)
C9B	0.0263 (6)	0.0535 (10)	0.1226 (18)	−0.0024 (6)	−0.0072 (8)	0.0030 (11)
C10B	0.0358 (6)	0.0266 (5)	0.0306 (6)	0.0012 (4)	−0.0068 (4)	0.0051 (4)
C11B	0.0206 (4)	0.0276 (5)	0.0199 (4)	0.0015 (4)	−0.0020 (3)	−0.0019 (4)
O5B	0.044 (3)	0.0460 (17)	0.025 (2)	0.0018 (18)	−0.0094 (18)	−0.0140 (14)
N1B	0.0313 (19)	0.034 (2)	0.0166 (19)	0.0050 (12)	0.0028 (10)	−0.0165 (13)
C12B	0.0168 (14)	0.039 (3)	0.022 (2)	0.0082 (14)	−0.0033 (13)	−0.0089 (15)
C13B	0.0186 (16)	0.0277 (16)	0.0168 (14)	0.0033 (13)	−0.0052 (14)	−0.0116 (11)
C14B	0.0250 (14)	0.036 (2)	0.0273 (17)	0.0022 (14)	−0.0035 (11)	−0.0064 (16)
C15B	0.0246 (9)	0.0413 (12)	0.0254 (9)	−0.0051 (8)	−0.0078 (7)	−0.0024 (9)
C16B	0.0154 (13)	0.0386 (19)	0.0245 (18)	0.0026 (12)	−0.0049 (11)	0.0001 (14)
C17B	0.0198 (8)	0.0370 (11)	0.0216 (9)	0.0018 (7)	−0.0014 (7)	−0.0058 (8)
C18B	0.0415 (12)	0.0370 (12)	0.0367 (12)	0.0060 (9)	0.0004 (9)	0.0069 (9)
C19B	0.0224 (9)	0.0731 (19)	0.0351 (12)	0.0124 (10)	−0.0029 (8)	−0.0055 (12)
O5C	0.039 (2)	0.059 (2)	0.026 (3)	−0.0141 (15)	0.0007 (16)	−0.0192 (19)
N1C	0.0102 (13)	0.025 (2)	0.012 (2)	0.0063 (12)	−0.0053 (14)	−0.0123 (18)
C12C	0.028 (2)	0.035 (2)	0.0154 (18)	0.0042 (16)	−0.0047 (15)	−0.0139 (15)
C13C	0.024 (2)	0.029 (2)	0.028 (2)	0.0077 (15)	0.0039 (15)	−0.0020 (16)
C14C	0.034 (2)	0.039 (3)	0.0197 (18)	−0.0018 (18)	−0.0065 (15)	−0.009 (2)
C15C	0.0299 (12)	0.0471 (16)	0.0252 (12)	0.0027 (11)	−0.0088 (9)	−0.0059 (11)
C16C	0.029 (2)	0.035 (2)	0.024 (2)	0.0004 (15)	−0.0035 (15)	−0.0040 (16)
C17C	0.0234 (10)	0.0253 (11)	0.0246 (11)	0.0024 (9)	−0.0036 (8)	−0.0028 (9)
C18C	0.0343 (14)	0.0524 (18)	0.0434 (17)	0.0172 (13)	−0.0090 (12)	−0.0012 (14)
C19C	0.0297 (12)	0.0467 (16)	0.0356 (14)	−0.0080 (11)	0.0034 (10)	−0.0072 (12)

### 2-Chloro-N-(5,5-dimethyl-3-oxocyclohex-1-en-1-yl)-4-ethoxy-3,5-dimethoxybenzamide (3b) Geometric parameters (Å, º)

**Table:**

Cl1A—C2A	1.7331 (12)	C3B—C4B	1.3908 (15)
O1A—C3A	1.3684 (14)	C4B—C5B	1.4042 (15)
O1A—C7A	1.4281 (17)	C5B—C6B	1.3903 (14)
O2A—C4A	1.3678 (13)	C6B—H6BA	0.9500
O2A—C8A	1.4381 (16)	C7B—H7BA	0.9800
O3A—C5A	1.3667 (15)	C7B—H7BB	0.9800
O3A—C10A	1.4313 (19)	C7B—H7BC	0.9800
O4A—C11A	1.2163 (13)	C8B—C9B	1.502 (2)
O5A—C14A	1.2349 (13)	C8B—H8BA	0.9900
N1A—C11A	1.3722 (13)	C8B—H8BB	0.9900
N1A—C12A	1.3859 (12)	C9B—H9BA	0.9800
N1A—H1AA	0.853 (16)	C9B—H9BB	0.9800
C1A—C2A	1.3885 (15)	C9B—H9BC	0.9800
C1A—C6A	1.3964 (15)	C10B—H10D	0.9800
C1A—C11A	1.5047 (14)	C10B—H10E	0.9800
C2A—C3A	1.4044 (15)	C10B—H10F	0.9800
C3A—C4A	1.3956 (17)	C11B—N1B	1.330 (8)
C4A—C5A	1.4053 (17)	C11B—N1C	1.421 (8)
C5A—C6A	1.3886 (15)	O5B—C14B	1.229 (8)
C6A—H6AA	0.9500	N1B—C12B	1.384 (8)
C7A—H7AA	0.9800	N1B—H1BA	0.77 (5)
C7A—H7AB	0.9800	C12B—C13B	1.330 (7)
C7A—H7AC	0.9800	C12B—C17B	1.545 (8)
C8A—C9A	1.480 (2)	C13B—C14B	1.455 (7)
C8A—H8AA	0.9900	C13B—H13B	0.9500
C8A—H8AB	0.9900	C14B—C15B	1.515 (8)
C9A—H9AA	0.9800	C15B—C16B	1.535 (7)
C9A—H9AB	0.9800	C15B—H15B	0.9900
C9A—H9AC	0.9800	C15B—H15D	0.9900
C10A—H10A	0.9800	C16B—C19B	1.530 (7)
C10A—H10B	0.9800	C16B—C18B	1.532 (8)
C10A—H10C	0.9800	C16B—C17B	1.540 (6)
C12A—C13A	1.3501 (14)	C17B—H17C	0.9900
C12A—C17A	1.5001 (15)	C17B—H17D	0.9900
C13A—C14A	1.4486 (14)	C18B—H18D	0.9800
C13A—H13A	0.9500	C18B—H18E	0.9800
C14A—C15A	1.4989 (16)	C18B—H18F	0.9800
C15A—C16A	1.5351 (18)	C19B—H19D	0.9800
C15A—H15A	0.9900	C19B—H19E	0.9800
C15A—H15C	0.9900	C19B—H19F	0.9800
C16A—C19A	1.519 (2)	O5C—C14C	1.244 (9)
C16A—C18A	1.5263 (19)	N1C—C12C	1.401 (9)
C16A—C17A	1.5364 (16)	N1C—H1CA	0.88 (5)
C17A—H17A	0.9900	C12C—C13C	1.342 (9)
C17A—H17B	0.9900	C12C—C17C	1.527 (10)
C18A—H18A	0.9800	C13C—C14C	1.450 (9)
C18A—H18B	0.9800	C13C—H13C	0.9500
C18A—H18C	0.9800	C14C—C15C	1.499 (8)
C19A—H19A	0.9800	C15C—C16C	1.532 (9)
C19A—H19B	0.9800	C15C—H15E	0.9900
C19A—H19C	0.9800	C15C—H15F	0.9900
Cl1B—C2B	1.7334 (11)	C16C—C17C	1.528 (9)
O1B—C3B	1.3715 (13)	C16C—C19C	1.537 (10)
O1B—C7B	1.4379 (17)	C16C—C18C	1.545 (9)
O2B—C4B	1.3724 (12)	C17C—H17E	0.9900
O2B—C8B	1.4510 (16)	C17C—H17F	0.9900
O3B—C5B	1.3605 (13)	C18C—H18G	0.9800
O3B—C10B	1.4277 (14)	C18C—H18H	0.9800
O4B—C11B	1.2147 (13)	C18C—H18I	0.9800
C1B—C2B	1.3891 (15)	C19C—H19G	0.9800
C1B—C6B	1.3986 (14)	C19C—H19H	0.9800
C1B—C11B	1.5065 (14)	C19C—H19I	0.9800
C2B—C3B	1.4000 (14)		
			
C3A—O1A—C7A	114.34 (10)	O1B—C7B—H7BB	109.5
C4A—O2A—C8A	116.92 (10)	H7BA—C7B—H7BB	109.5
C5A—O3A—C10A	116.80 (10)	O1B—C7B—H7BC	109.5
C11A—N1A—C12A	127.99 (9)	H7BA—C7B—H7BC	109.5
C11A—N1A—H1AA	118.9 (11)	H7BB—C7B—H7BC	109.5
C12A—N1A—H1AA	112.8 (11)	O2B—C8B—C9B	112.15 (12)
C2A—C1A—C6A	119.60 (10)	O2B—C8B—H8BA	109.2
C2A—C1A—C11A	124.39 (10)	C9B—C8B—H8BA	109.2
C6A—C1A—C11A	115.99 (9)	O2B—C8B—H8BB	109.2
C1A—C2A—C3A	120.34 (10)	C9B—C8B—H8BB	109.2
C1A—C2A—Cl1A	121.28 (8)	H8BA—C8B—H8BB	107.9
C3A—C2A—Cl1A	118.34 (9)	C8B—C9B—H9BA	109.5
O1A—C3A—C4A	119.96 (10)	C8B—C9B—H9BB	109.5
O1A—C3A—C2A	119.96 (11)	H9BA—C9B—H9BB	109.5
C4A—C3A—C2A	119.94 (10)	C8B—C9B—H9BC	109.5
O2A—C4A—C3A	117.42 (11)	H9BA—C9B—H9BC	109.5
O2A—C4A—C5A	122.99 (11)	H9BB—C9B—H9BC	109.5
C3A—C4A—C5A	119.50 (10)	O3B—C10B—H10D	109.5
O3A—C5A—C6A	124.09 (11)	O3B—C10B—H10E	109.5
O3A—C5A—C4A	115.87 (10)	H10D—C10B—H10E	109.5
C6A—C5A—C4A	120.03 (11)	O3B—C10B—H10F	109.5
C5A—C6A—C1A	120.56 (11)	H10D—C10B—H10F	109.5
C5A—C6A—H6AA	119.7	H10E—C10B—H10F	109.5
C1A—C6A—H6AA	119.7	O4B—C11B—N1B	120.3 (4)
O1A—C7A—H7AA	109.5	O4B—C11B—N1C	127.6 (4)
O1A—C7A—H7AB	109.5	O4B—C11B—C1B	120.55 (9)
H7AA—C7A—H7AB	109.5	N1B—C11B—C1B	119.1 (4)
O1A—C7A—H7AC	109.5	N1C—C11B—C1B	111.8 (4)
H7AA—C7A—H7AC	109.5	C11B—N1B—C12B	131.6 (8)
H7AB—C7A—H7AC	109.5	C11B—N1B—H1BA	115 (4)
O2A—C8A—C9A	110.05 (14)	C12B—N1B—H1BA	112 (4)
O2A—C8A—H8AA	109.7	C13B—C12B—N1B	122.2 (7)
C9A—C8A—H8AA	109.7	C13B—C12B—C17B	123.6 (6)
O2A—C8A—H8AB	109.7	N1B—C12B—C17B	113.1 (6)
C9A—C8A—H8AB	109.7	C12B—C13B—C14B	119.3 (6)
H8AA—C8A—H8AB	108.2	C12B—C13B—H13B	120.4
C8A—C9A—H9AA	109.5	C14B—C13B—H13B	120.4
C8A—C9A—H9AB	109.5	O5B—C14B—C13B	120.7 (7)
H9AA—C9A—H9AB	109.5	O5B—C14B—C15B	119.7 (7)
C8A—C9A—H9AC	109.5	C13B—C14B—C15B	119.6 (6)
H9AA—C9A—H9AC	109.5	C14B—C15B—C16B	113.2 (4)
H9AB—C9A—H9AC	109.5	C14B—C15B—H15B	108.9
O3A—C10A—H10A	109.5	C16B—C15B—H15B	108.9
O3A—C10A—H10B	109.5	C14B—C15B—H15D	108.9
H10A—C10A—H10B	109.5	C16B—C15B—H15D	108.9
O3A—C10A—H10C	109.5	H15B—C15B—H15D	107.8
H10A—C10A—H10C	109.5	C19B—C16B—C18B	109.4 (4)
H10B—C10A—H10C	109.5	C19B—C16B—C15B	109.7 (4)
O4A—C11A—N1A	124.73 (9)	C18B—C16B—C15B	110.3 (4)
O4A—C11A—C1A	121.52 (9)	C19B—C16B—C17B	109.4 (4)
N1A—C11A—C1A	113.66 (9)	C18B—C16B—C17B	110.3 (4)
C13A—C12A—N1A	124.56 (9)	C15B—C16B—C17B	107.8 (4)
C13A—C12A—C17A	122.20 (9)	C16B—C17B—C12B	111.3 (4)
N1A—C12A—C17A	113.25 (9)	C16B—C17B—H17C	109.4
C12A—C13A—C14A	121.19 (10)	C12B—C17B—H17C	109.4
C12A—C13A—H13A	119.4	C16B—C17B—H17D	109.4
C14A—C13A—H13A	119.4	C12B—C17B—H17D	109.4
O5A—C14A—C13A	121.13 (10)	H17C—C17B—H17D	108.0
O5A—C14A—C15A	120.98 (10)	C16B—C18B—H18D	109.5
C13A—C14A—C15A	117.88 (9)	C16B—C18B—H18E	109.5
C14A—C15A—C16A	112.84 (9)	H18D—C18B—H18E	109.5
C14A—C15A—H15A	109.0	C16B—C18B—H18F	109.5
C16A—C15A—H15A	109.0	H18D—C18B—H18F	109.5
C14A—C15A—H15C	109.0	H18E—C18B—H18F	109.5
C16A—C15A—H15C	109.0	C16B—C19B—H19D	109.5
H15A—C15A—H15C	107.8	C16B—C19B—H19E	109.5
C19A—C16A—C18A	110.90 (16)	H19D—C19B—H19E	109.5
C19A—C16A—C15A	110.13 (12)	C16B—C19B—H19F	109.5
C18A—C16A—C15A	108.85 (12)	H19D—C19B—H19F	109.5
C19A—C16A—C17A	110.10 (11)	H19E—C19B—H19F	109.5
C18A—C16A—C17A	108.99 (12)	C12C—N1C—C11B	123.0 (9)
C15A—C16A—C17A	107.80 (11)	C12C—N1C—H1CA	119 (3)
C12A—C17A—C16A	113.03 (9)	C11B—N1C—H1CA	117 (3)
C12A—C17A—H17A	109.0	C13C—C12C—N1C	128.2 (9)
C16A—C17A—H17A	109.0	C13C—C12C—C17C	118.0 (8)
C12A—C17A—H17B	109.0	N1C—C12C—C17C	111.9 (7)
C16A—C17A—H17B	109.0	C12C—C13C—C14C	124.8 (8)
H17A—C17A—H17B	107.8	C12C—C13C—H13C	117.6
C16A—C18A—H18A	109.5	C14C—C13C—H13C	117.6
C16A—C18A—H18B	109.5	O5C—C14C—C13C	119.6 (9)
H18A—C18A—H18B	109.5	O5C—C14C—C15C	123.6 (8)
C16A—C18A—H18C	109.5	C13C—C14C—C15C	116.8 (7)
H18A—C18A—H18C	109.5	C14C—C15C—C16C	113.6 (5)
H18B—C18A—H18C	109.5	C14C—C15C—H15E	108.8
C16A—C19A—H19A	109.5	C16C—C15C—H15E	108.8
C16A—C19A—H19B	109.5	C14C—C15C—H15F	108.8
H19A—C19A—H19B	109.5	C16C—C15C—H15F	108.8
C16A—C19A—H19C	109.5	H15E—C15C—H15F	107.7
H19A—C19A—H19C	109.5	C17C—C16C—C15C	109.0 (6)
H19B—C19A—H19C	109.5	C17C—C16C—C19C	110.0 (6)
C3B—O1B—C7B	112.62 (9)	C15C—C16C—C19C	110.6 (6)
C4B—O2B—C8B	114.21 (10)	C17C—C16C—C18C	108.9 (6)
C5B—O3B—C10B	116.84 (9)	C15C—C16C—C18C	109.4 (6)
C2B—C1B—C6B	119.81 (9)	C19C—C16C—C18C	109.0 (6)
C2B—C1B—C11B	123.40 (9)	C12C—C17C—C16C	112.2 (6)
C6B—C1B—C11B	116.68 (9)	C12C—C17C—H17E	109.2
C1B—C2B—C3B	120.05 (9)	C16C—C17C—H17E	109.2
C1B—C2B—Cl1B	121.83 (8)	C12C—C17C—H17F	109.2
C3B—C2B—Cl1B	118.10 (8)	C16C—C17C—H17F	109.2
O1B—C3B—C4B	119.85 (9)	H17E—C17C—H17F	107.9
O1B—C3B—C2B	120.07 (10)	C16C—C18C—H18G	109.5
C4B—C3B—C2B	120.08 (10)	C16C—C18C—H18H	109.5
O2B—C4B—C3B	120.17 (10)	H18G—C18C—H18H	109.5
O2B—C4B—C5B	119.75 (10)	C16C—C18C—H18I	109.5
C3B—C4B—C5B	119.98 (9)	H18G—C18C—H18I	109.5
O3B—C5B—C6B	125.11 (10)	H18H—C18C—H18I	109.5
O3B—C5B—C4B	115.33 (9)	C16C—C19C—H19G	109.5
C6B—C5B—C4B	119.56 (10)	C16C—C19C—H19H	109.5
C5B—C6B—C1B	120.46 (10)	H19G—C19C—H19H	109.5
C5B—C6B—H6BA	119.8	C16C—C19C—H19I	109.5
C1B—C6B—H6BA	119.8	H19G—C19C—H19I	109.5
O1B—C7B—H7BA	109.5	H19H—C19C—H19I	109.5
			
C6A—C1A—C2A—C3A	0.43 (16)	C8B—O2B—C4B—C3B	79.16 (14)
C11A—C1A—C2A—C3A	−177.91 (10)	C8B—O2B—C4B—C5B	−104.32 (12)
C6A—C1A—C2A—Cl1A	−177.38 (8)	O1B—C3B—C4B—O2B	−3.90 (16)
C11A—C1A—C2A—Cl1A	4.27 (15)	C2B—C3B—C4B—O2B	177.06 (9)
C7A—O1A—C3A—C4A	−89.70 (15)	O1B—C3B—C4B—C5B	179.58 (9)
C7A—O1A—C3A—C2A	94.67 (14)	C2B—C3B—C4B—C5B	0.54 (16)
C1A—C2A—C3A—O1A	176.11 (10)	C10B—O3B—C5B—C6B	0.54 (16)
Cl1A—C2A—C3A—O1A	−6.00 (14)	C10B—O3B—C5B—C4B	−179.34 (10)
C1A—C2A—C3A—C4A	0.48 (16)	O2B—C4B—C5B—O3B	0.86 (15)
Cl1A—C2A—C3A—C4A	178.36 (9)	C3B—C4B—C5B—O3B	177.39 (10)
C8A—O2A—C4A—C3A	124.66 (15)	O2B—C4B—C5B—C6B	−179.03 (10)
C8A—O2A—C4A—C5A	−58.83 (18)	C3B—C4B—C5B—C6B	−2.50 (16)
O1A—C3A—C4A—O2A	0.97 (16)	O3B—C5B—C6B—C1B	−177.06 (10)
C2A—C3A—C4A—O2A	176.61 (10)	C4B—C5B—C6B—C1B	2.82 (15)
O1A—C3A—C4A—C5A	−175.67 (10)	C2B—C1B—C6B—C5B	−1.17 (15)
C2A—C3A—C4A—C5A	−0.03 (17)	C11B—C1B—C6B—C5B	−177.57 (9)
C10A—O3A—C5A—C6A	−3.11 (19)	C4B—O2B—C8B—C9B	67.92 (16)
C10A—O3A—C5A—C4A	178.00 (13)	C2B—C1B—C11B—O4B	−122.81 (13)
O2A—C4A—C5A—O3A	1.15 (17)	C6B—C1B—C11B—O4B	53.44 (14)
C3A—C4A—C5A—O3A	177.60 (10)	C2B—C1B—C11B—N1B	58.5 (6)
O2A—C4A—C5A—C6A	−177.78 (10)	C6B—C1B—C11B—N1B	−125.2 (6)
C3A—C4A—C5A—C6A	−1.34 (17)	C2B—C1B—C11B—N1C	59.4 (5)
O3A—C5A—C6A—C1A	−176.57 (11)	C6B—C1B—C11B—N1C	−124.3 (5)
C4A—C5A—C6A—C1A	2.27 (17)	O4B—C11B—N1B—C12B	7.2 (16)
C2A—C1A—C6A—C5A	−1.81 (16)	C1B—C11B—N1B—C12B	−174.2 (11)
C11A—C1A—C6A—C5A	176.67 (10)	C11B—N1B—C12B—C13B	0 (2)
C4A—O2A—C8A—C9A	−148.77 (16)	C11B—N1B—C12B—C17B	−169.0 (10)
C12A—N1A—C11A—O4A	−2.59 (18)	N1B—C12B—C13B—C14B	−175.2 (12)
C12A—N1A—C11A—C1A	174.03 (10)	C17B—C12B—C13B—C14B	−7.6 (18)
C2A—C1A—C11A—O4A	−129.47 (12)	C12B—C13B—C14B—O5B	−173.3 (12)
C6A—C1A—C11A—O4A	52.13 (15)	C12B—C13B—C14B—C15B	8.2 (15)
C2A—C1A—C11A—N1A	53.79 (14)	O5B—C14B—C15B—C16B	148.7 (9)
C6A—C1A—C11A—N1A	−124.61 (10)	C13B—C14B—C15B—C16B	−32.8 (11)
C11A—N1A—C12A—C13A	−9.78 (18)	C14B—C15B—C16B—C19B	172.5 (5)
C11A—N1A—C12A—C17A	170.04 (11)	C14B—C15B—C16B—C18B	−66.9 (6)
N1A—C12A—C13A—C14A	−176.55 (10)	C14B—C15B—C16B—C17B	53.5 (7)
C17A—C12A—C13A—C14A	3.65 (17)	C19B—C16B—C17B—C12B	−170.7 (7)
C12A—C13A—C14A—O5A	173.40 (12)	C18B—C16B—C17B—C12B	68.9 (7)
C12A—C13A—C14A—C15A	−8.11 (17)	C15B—C16B—C17B—C12B	−51.5 (7)
O5A—C14A—C15A—C16A	−146.37 (12)	C13B—C12B—C17B—C16B	31.0 (15)
C13A—C14A—C15A—C16A	35.13 (16)	N1B—C12B—C17B—C16B	−160.4 (9)
C14A—C15A—C16A—C19A	65.22 (14)	O4B—C11B—N1C—C12C	9.7 (15)
C14A—C15A—C16A—C18A	−173.01 (13)	C1B—C11B—N1C—C12C	−172.7 (11)
C14A—C15A—C16A—C17A	−54.92 (14)	C11B—N1C—C12C—C13C	−8 (3)
C13A—C12A—C17A—C16A	−26.27 (17)	C11B—N1C—C12C—C17C	155.4 (10)
N1A—C12A—C17A—C16A	153.90 (11)	N1C—C12C—C13C—C14C	176.1 (15)
C19A—C16A—C17A—C12A	−70.04 (15)	C17C—C12C—C13C—C14C	13 (2)
C18A—C16A—C17A—C12A	168.10 (14)	C12C—C13C—C14C—O5C	171.1 (16)
C15A—C16A—C17A—C12A	50.11 (15)	C12C—C13C—C14C—C15C	−11 (2)
C6B—C1B—C2B—C3B	−0.81 (14)	O5C—C14C—C15C—C16C	−151.4 (13)
C11B—C1B—C2B—C3B	175.34 (9)	C13C—C14C—C15C—C16C	30.3 (14)
C6B—C1B—C2B—Cl1B	−178.96 (8)	C14C—C15C—C16C—C17C	−51.9 (9)
C11B—C1B—C2B—Cl1B	−2.82 (14)	C14C—C15C—C16C—C19C	69.1 (9)
C7B—O1B—C3B—C4B	79.27 (13)	C14C—C15C—C16C—C18C	−170.8 (7)
C7B—O1B—C3B—C2B	−101.69 (13)	C13C—C12C—C17C—C16C	−35.8 (17)
C1B—C2B—C3B—O1B	−177.93 (9)	N1C—C12C—C17C—C16C	158.7 (11)
Cl1B—C2B—C3B—O1B	0.30 (14)	C15C—C16C—C17C—C12C	53.9 (10)
C1B—C2B—C3B—C4B	1.12 (15)	C19C—C16C—C17C—C12C	−67.5 (9)
Cl1B—C2B—C3B—C4B	179.34 (8)	C18C—C16C—C17C—C12C	173.2 (8)

### 2-Chloro-N-(5,5-dimethyl-3-oxocyclohex-1-en-1-yl)-4-ethoxy-3,5-dimethoxybenzamide (3b) Hydrogen-bond geometry (Å, º)

**Table:**

*D*—H···*A*	*D*—H	H···*A*	*D*···*A*	*D*—H···*A*
N1*A*—H1*AA*···O5*B*	0.853 (16)	2.040 (18)	2.849 (8)	158.1 (15)
N1*A*—H1*AA*···O5*C*	0.853 (16)	2.081 (19)	2.909 (9)	163.5 (15)
C7*A*—H7*AA*···O2*B*^i^	0.98	2.44	3.3451 (17)	153
C8*A*—H8*AB*···Cl1*A*^ii^	0.99	2.92	3.703 (2)	137
C17*A*—H17*A*···O5*B*	0.99	2.42	3.285 (7)	146
C17*A*—H17*A*···O5*C*	0.99	2.63	3.481 (8)	144
C10*B*—H10*F*···O4*A*^iii^	0.98	2.46	3.4013 (15)	161
N1*B*—H1*BA*···O5*A*^iv^	0.77 (5)	2.31 (5)	2.985 (10)	147 (5)
N1*C*—H1*CA*···O5*A*^iv^	0.88 (5)	1.96 (4)	2.795 (12)	159 (4)
C17*C*—H17*E*···O5*A*^iv^	0.99	2.54	3.364 (3)	141

Symmetry codes: (i) −*x*+1, *y*+1/2, −*z*+1/2; (ii) −*x*+2, −*y*+2, −*z*+1; (iii) −*x*+1, −*y*+2, −*z*+1; (iv) *x*, −*y*+3/2, *z*−1/2.
